# Prevalence and predictors of alcohol and drug use among secondary school students in Botswana: a cross-sectional study

**DOI:** 10.1186/s12889-018-6263-2

**Published:** 2018-12-20

**Authors:** Katherine Riva, Lynne Allen-Taylor, Will D. Schupmann, Seipone Mphele, Neo Moshashane, Elizabeth D. Lowenthal

**Affiliations:** 1 Department of Psychiatry, University of Pennsylvania Perelman School of Medicine, 3535 Market Street, Second Floor, Philadelphia, PA 19104 USA; 2 Center for Clinical Epidemiology and Biostatistics, University of Pennsylvania Perelman School of Medicine, 516B Blockley Hall, 423 Guardian Drive, Philadelphia, PA 19104 USA; 3 University of Pennsylvania Perelman School of Medicine, 2716 South St, Room 11242, Philadelphia, PA 19146 USA; 4 Department of Psychology, University of Botswana, Private Bag UB 0022, Gaborone, Botswana; 5 Pediatrics and Epidemiology, University of Pennsylvania Perelman School of Medicine, 2716 South St, Room 11242, Philadelphia, PA 19146 USA

**Keywords:** Africa, Botswana, Secondary School, Student, Adolescent, Youth, Alcohol, Drugs, Risk factors, Protective factors

## Abstract

**Background:**

Alcohol and illicit drug use has been recognized as a growing problem among adolescents in Botswana. Little is known about factors affecting alcohol and drug use among Botswana’s secondary school students. To aid the design and implementation of effective public health interventions, we sought to determine the prevalence of alcohol and drug use in secondary school students in urban and peri-urban areas of Botswana, and to evaluate risk and protective factors for substance use.

**Methods:**

We performed a 72-item cross-sectional survey of students in 17 public secondary schools in Gaborone, Lobatse, Molepolole and Mochudi, Botswana. The World Health Organization’s (WHO) Alcohol Use Disorder Identification Test (AUDIT) was used to define hazardous drinking behavior. Using Jessor’s Problem Behavior Theory (PBT) as our conceptual framework, we culturally-adapted items from previously validated tools to measure risk and protective factors for alcohol and drug use. Between-group differences of risk and protective factors were compared using univariate binomial and multinomial-ordinal logit analysis. Relative risks of alcohol and drug use by demographic, high risks and low protections were calculated. Multivariate ordinal-multinomial cumulative logit analysis, multivariate nominal-multinomial logit analysis, and binominal logit analysis were used to build models illustrating the relationship between risk and protective factors and student alcohol and illicit drug use. Clustered data was adjusted for in all analyses using Generalized Estimating Equations (GEE) methods.

**Results:**

Of the 1936 students surveyed, 816 (42.1%) reported alcohol use, and 434 (22.4%) met criteria for hazardous alcohol use. Illicit drug use was reported by 324 students (16.7%), with motokwane (marijuana) being the most commonly used drug. Risk factors more strongly associated with alcohol and drug use were reported alcohol availability, individual and social vulnerability factors, and poor peer modeling. Individual and social controls protections appear to mitigate risk of student alcohol and drug use.

**Conclusions:**

Alcohol and illicit drug use is prevalent among secondary school students in Botswana. Our data suggest that interventions that reduce the availability of alcohol and drugs and that build greater support networks for adolescents may be most helpful in decreasing alcohol and drug use among secondary school students in Botswana.

**Electronic supplementary material:**

The online version of this article (10.1186/s12889-018-6263-2) contains supplementary material, which is available to authorized users.

## Background

Recent studies have shown that alcohol and illicit drug use is prevalent among secondary school students in Botswana. In 2005, the WHO-developed Global School-based Health Survey (GSHS) showed that 20.9% of surveyed 13- to 15-year-old secondary school students in Botswana “drank so much alcohol that they were really drunk one or more times,” and 7.5% used illicit drugs one or more times during their lifetime [1]. The 2010 Youth Risk Behaviour Surveillance Survey conducted by the Botswana Ministry of Education and Skills Development (MOE) showed that 37.5% of students who reported alcohol use had their first drink before the age of 13 and 13.2% of the 3567 students surveyed had used marijuana during their lifetime [2].

Alcohol use at an early age is a concern due to its negative effects on the health, wellbeing, and development of young people. For example, students in several lower- to middle-income countries who have engaged in alcohol use are at higher risk for psychological distress [3]. Alcohol use in early adolescence is also associated with a higher risk of developing mental health disorders and alcohol-related problems later in life [4]. Alcohol and drug use is also associated with an increase in other risky behaviors such as early sexual debut, unprotected sex, drunk driving, violence and truancy [5–9].

Factors affecting alcohol and drug use among adolescents in different contexts are numerous and complex. In this study, we set forth to examine risk and protective factors for alcohol and drug use among secondary school students in Botswana using Jessor’s Problem Behavior Theory (PBT) as a conceptual framework. Jessor’s PBT is a cross-culturally validated model that explains problem behaviors among adolescents, including alcohol and drug use [6]. PBT was reorganized in recent years to describe adolescent health behavior in terms of risk and protective constructs at the individual, family, and community levels [10]. In this model, three domains of protection (models, controls and support) and three domains of risk (models, opportunity and vulnerability) account for the variability in adolescent health behaviors. (See Method section for definitions of protection and risk domains). We chose this model as our framework to identify and examine culturally-specific risk and protective factors for alcohol and drug use among secondary school students in Botswana because of its relevance to the question of interest and because it has been validated in sub-Saharan Africa [11].

Understanding the common risk and protective factors associated with alcohol and drug use among secondary school students is imperative to the design and implementation of effective public health interventions. By furthering understanding of these issues, we hope to inform and promote the design of effective student alcohol and drug use prevention programs in this and similar settings.

## Methods

### Participants and procedures

A 72-item cross-sectional survey was administered to secondary school students at 17 secondary schools in Botswana’s capital city, Gaborone, and surrounding villages Lobatse, Molepolole, and Mochudi. We included all seven public senior secondary schools in our target area, and randomly selected ten of the 29 public junior secondary schools in blocks to include six schools in Gaborone, two in Molepolole and one each in of the smaller communities of Lobatse and Mochudi.

All students at these schools have study hall periods during which they can complete work for other classes. The Botswana Ministry of Education recommended that these periods be used for study data collection. At each junior secondary school, one study hall classroom per grade level (grades include Form 1 to 3, equivalent to grades 8 to 10 at American schools) was randomly selected to participate in the survey. At each senior secondary school, two Form 4 study hall classrooms and one Form 5 study hall classroom were randomly selected.

Surveys were administered from January 2013 to March 2013. English is the formal language of instruction in public secondary schools in Botswana. However, some students are more comfortable with the local native language, Setswana. Students were informed of the study in both Setswana and English, and their assent was demonstrated through voluntary participation in the survey. Answer sheets were labeled with unique study identification (ID) numbers instead of student names to ensure the confidentiality of student responses, and students were reassured that school officials, teachers, and parents would not have access to their survey responses. Study ID numbers were linked to names on a separate protected list, to allow for Institutional Review Board-approved follow-up interviews and focus group discussions based on responses. Teachers and school administrators were not present in the classroom while the surveys were completed. The surveys were administered during one hour-long study hall period by trained research assistants from University of Botswana, and were available in both English and Setswana.

### Survey instrument and study variables

The written survey consisted of 72-items including: 1) demographic characteristics (3 items), 2) alcohol use (10 items), and 3) drug use (4 items), risk and protective factors for alcohol and drug use (43 items), and 5) motivations for alcohol use (12 items).

#### Primary outcome variables

Alcohol use was measured with the Alcohol Use and Dependency Inventory Tool (AUDIT) [13]. AUDIT is a WHO-developed screening tool and is cross-culturally validated in sub-Saharan Africa [13, 14]. The 10-item survey focuses on recent alcohol use, and includes questions regarding hazardous alcohol use and symptoms of alcohol dependency. In adults, an AUDIT score of 8 or greater indicates hazardous drinking habits and possible alcohol dependency [14]. However, multiple studies recommend a lower cut-off score to identify problematic or hazardous alcohol use in adolescent populations [15–19]. In analyzing the data, we trichotomized AUDIT scores as follows: non-drinkers (AUDIT score = zero), lower-risk drinkers (AUDIT score 1 to 4), and hazardous drinkers (AUDIT score ≥ 5) [14–19].

Drug use was measured through survey items from the Botswana version of WHO’s Global Student Health Survey (GSHS). Items measured frequency of drug use during students’ lifetime, past year and past 30 days, as well as the type of drug used most often. We defined drug use in our survey as use of a drug such as marijuana, mandrax, cocaine, ecstasy, glue, benzene, methanol, prescription drugs not prescribed by a doctor, or other. Student drug use was defined as any admission of illicit drug use.

#### Risk factors

Risk Factors for alcohol and drug use were measured in three domains: Models Risk (family alcohol use, peer alcohol use), Opportunity Risk (availability of alcohol at home, social gatherings, and in the community), and Vulnerability Risks (family tension, peer tension, school tension, low self-perception, low expectations for the future, suicidal ideation). Models Risk and the Vulnerability Risks of low self-perception and low expectations for the future were measured using survey items developed and used to test the validity of the PBT model in Nairobi, Kenya [11]. We measured opportunity risk with four items: “If you wanted alcohol to drink, would you be able to get some at home?” [10]. “How many times have you lied about your age to buy alcohol?” [12], “How many times has someone bought alcohol for you?” and “How many times has someone offered you alcohol at a party or wedding?” Family, peer and school level vulnerability were each measured with a single item [10]. One item to assess depressive symptoms/suicidality was added from the GSHS Botswana survey, “During the past 12 months, did you ever seriously consider attempting suicide?”

#### Protective factors

The survey measured protective factors in three domains: Control Protection (parental control, peer control, school attachment, and religiosity), Support Protection (parental support, teacher support, peer support) and Models Protection (positive peer modeling). The majority of survey items were adopted from the survey tool developed and used to test the validity of Jessor’s PBT model in Nairobi, Kenya [11]. An item from the 2005 Botswana GSHS was included to assess peer support, a variable not measured in the Kenyan study. See Additional file 1: Table S1 for more details on survey items measuring risk and protective factors.

### Risk and protective factor composite and subcomponent scores

Ordinal protective and risk factor survey items were standardized using z-scores (mean = 0, standard deviation = 1), in line with other studies that have evaluated adolescent problem behavior using the PBT framework [11, 20]. Composite and subcomponent scores were then created from the standardized items. Risks and protective factor subcomponent and composite scores were created from the standardized items based on 1) Jessor’s theoretical model of adolescent problem behavior, 2) item psychometrics such as coefficient alpha, inter-item correlations and partial correlations, 3) the Kaiser’s Measure of Sampling Adequacy (MSA), and 4) Principal Component Factor Analysis (eigenvalues > 1, Factor loadings > 0.60, varimax rotation as appropriate) [20–22]. Subsets of items designed to tap respective behaviors identified in the theoretical framework were first examined for internal consistency. The subset of items having 1) a coefficient alpha > 0.60, 2) low to moderate inter-item correlations and partial correlations, 3) overall MSA > 0.50, and 4) items loadings > 0.60 on the same factors that had an eigenvalue > 1, were considered internally consistent, homogenous, and measuring an underlying latent trait or construct. For the subset of homogeneous items, we computed the average of the standardized items. This subset measure, which we call a “domain subcomponent,” represents a risk or protective factor construct. Items that were not internally consistent with the subset (i.e. coefficient alpha increased when the item was excluded, low inter-item correlation, and loaded < 0.60 on the factor analysis factors), represent a separate risk or protective factor within their respective domain. See Additional file 1: Tables S2 and S3 for more data related to the Principal Component Factor Analysis for risk and protective factor survey items.

Composite measures were computed for each domain. Because we do not consider the individual risk factors to be causally related to each other and do not believe that if one event occurred, the other will, we computed a composite measured by summing the risk factors or protective factors within each domain. Specifically, the domain composite measure is the average of the standardized risk factors included in each respective domain. Dichotomous risk and protective factor items were not included in the composite and subcomponent measures and were analyzed as separate risk/protective factors within their domain. Based on frequent student requests for clarification of survey question number 7, we dropped the question about parental scolding/reprimanding from the composite family monitoring variable and composite social controls protection variables.

### Secondary exposure variables

Composite and subcomponent risk and protective factor scores were also dichotomized to define high/low risk factor and high/low protective factor groups. “High risk” was defined as equal or greater than one standard deviation above the mean and “low protection” was defined by one or more standard deviation below the mean. These dichotomized scores were then used to calculate relative risk ratios to determine how substance use differed between students with higher risks and lower protections.

### Data sources and statistical methods

Double data entry from paper surveys into a REDCap database with automated matching to identify errors in data entry, ensured transcription accuracy [23]. Data were analyzed using STATA Data Analysis and Statistical Software version 12.1 and SAS/STAT 13.2 [24, 25].

### Design effect and effective sample size

Because the sampling design involved clustered data, students may not represent independent observations. Using a general linear nested model, we computed an intracluster correlation coefficient (ICC) to measure of the relatedness or similarity of our clustered data by comparing between cluster variability relative to between and within cluster variability. Design Effect and Effective sampling size were computed using ICC values.

### Univariate analysis

To examine the relationship between demographic variables and our two primary outcomes, 1) alcohol use by AUDIT score category (hazardous use, lower-risk use, and no alcohol use) and 2) drug use (yes, no) we computed summary statistics and conducted univariate binomial and multinomial-ordinal logit analyses. We used the Generalized Estimating Equations (GEE) method, specifying an independent correlation structure, to adjust for student-school clusters and obtain more efficient parameter estimates and better standard errors (See Table 1).

**Table 1:** Relationship between Demographic Variables and AUDIT Score Categories and Self-Reported Illicit Drug Use

	All study participants *N* = 1936			Alcohol Use *N* = 1927				Drug Use *N* = 1885		
		Hazardous Drinking *N* = 434 (22.4%)	Lower-risk Drinking *N* = 382 (19.7%)	Non-drinkers *N* = 1111 (57.4%)	Missing *N* = 9 (0.5%)	*p*-value*	Yes *N* = 324 (16.7%)	No *N* = 1561 (80.6%)	Missing *N* = 51 (2.6%)	*p*-value
	Median (IQR) or Number	Median (IQR) or *N* (%)	Median (IQR) or *N* (%)	Median (IQR) or *N* (%)	Median (IQR) or *N* (%)		Median (IQR) or *N* (%)	Median (IQR) or *N* (%)	Median (IQR) or *N* (%)	
Demographics**										
Age, *N* = 1935	16 (14–17) (mean 15.6)	16 (15–17) (mean 16.1)	15 (14–17) (mean 15.5)	16 (14–17) (mean 15.5)	15 (14–17) (mean 14.9)	0.003	16 (15–17) (mean 16.1)	16 (14–17) (mean 15.5)	15 (14–16) (mean 15.4)	< 0.001
Gender						0.003				
Male	904 (46.7%)	256 (28.3%)	180 (19.9%)	464 (51.3%)	4 (0.4%)		212 (23.5%)	671 (74.2%)	21 (2.3%)	< 0.001
Female	1029 (53.2%)	175 (17.0%)	202 (19.6%)	647 (62.9%)	5 (0.5%)		110 (10.7%)	889 (86.4%)	30 (2.9%)	
Missing	3 (0%)	3 (100%)	0 (0%)	0 (0%)	0 (0%)		2 (0.6%)	1 (0.1%)	0 (0%)	
Form in school (range 1–5)	3 (2–4) (mean 2.9)	3 (2–4) (mean 3.2)	3 (2–4) (mean 2.8)	3 (2–4) (mean 2.8)	2 (1–2) (mean 1.8)	0.005	4 (2–4) (mean 3.3)	3 (2–4) (mean 2.9)	2 (2–3) (mean 2.5)	< 0.001
Form 1	402 (20.8%)	63 (15.7%)	78 (19.4%)	257 (63.9%)	4 (1.0%)		43 (10.7%)	350 (87.1%)	9 (2.2%)	
Form 2	371 (19.2%)	68 (18.3%)	82 (22.1%)	218 (58.8%)	3 (0.8%)		44 (11.9%)	304 (81.9%)	23 (6.2%)	
Form 3	360 (18.6%)	87 (24.2%)	87 (24.2%)	185 (51.4%)	1 (0.2%)		64 (17.8%).	289 (80.3%)	7 (1.9%)	
Form 4	566 (29.2%)	131 (23.1%)	96 (17.0%)	338 (59.7%)	1 (0.2%)		107 (18.9%)	448 (79.2%)	11 (1.9%)	
Form 5	237 (12.2%)	85 (35.9%)	39 (16.4%)	113 (47.7%)	0 (0%)		66 (27.9%)	170 (71.7%)	1 (0.4%)	
School locations										
Urban	1173 (60.6%)	290 (24.7%)	233 (19.9%)	641 (54.6%)	9 (0.8%)	0.028	212 (18.1%)	928 (79.1%)	33 (2.8%)	0.24
Peri-urban	763 (39.4%)	144 (18.9%)	149 (19.5%)	470 (61.6%)	0 (0%)		112 (14.7%)	633 (83.0%)	18 (2.4%)	

Hazard drinking = AUDIT score “5+”, Low Risk Drinking is AUDIT scores “1,2,3,4”; Non-drinkers is AUDIT score “0” *Significance of differences between group analyzed using ordinal multinomial cumulative logit analysis using GEE methods to adjusted for clustering by school **Significance of differences between group analyzed using univariate binomial logit analysis using GEE methods to adjusted for clustering by school

Similarly, we examined the relationship between risk and protective measures and alcohol and drug use outcome variables by computing summary statistics and univariate binomial and multinomial-ordinal logit analysis using GEE methods to assess significance while taking into account clustered data (See Table 2).

**Table 2:** Differences in risk and protective factor measures by AUDIT categories and illicit drug use

		Hazardous Drinkers N = 434	Lower-risk Drinkers N = 382	Non-drinkers N = 1111	*p*-value*	Illicit drug use N = 324	No illicit drug use N = 1561	*p*-value*
	Mean (SD) or N (%)	Mean (SD) or N (%)	Mean (SD) or N (%)	Mean (SD) or N (%)		Mean (SD) or N (%)	Mean (SD) or N (%)	
Models Risk								
Sibling drinks alcohol	906 (47.4%)	259 (60.3%)	183 (48.3%)	463 (42.1%)	< 0.001	182 (57.1%)	709 (45.7%)	< 0.001
Problem drinker at home	680 (36.2%)	199 (47.3%)	141 (37.9%)	339 (31.3%)	<.0.001	154 (49.4%)	516 (33.8%)	< 0.001
Peer models risk (2 items)	0 (0.73)	0.43 (0.80)	−0.01 (0.72)	−0.16 (0.64)	< 0.001	0.35 (0.87)	−0.07 (0.68)	< 0.001
Vulnerability Risks								
Individual vulnerability risk (8 items)	−0.01 (0.58)	0.28 (0.67)	0.01 (0.54)	−0.13 (0.52)	< 0.001	0.36 (0.66)	−0.08 (0.53)	< 0.001
Social vulnerability risk (3 items)	0 (0.77)	0.33 (0.76)	0.03 (0.75)	−0.14 (0.74)	< 0.001	0.38 (0.78)	−0.07 (0.75)	< 0.001
Suicidal ideation (SI) in past year	381 (20.1%)	152 (36.3%)	75 (20.1%)	154 (14.0%)	< 0.001	118 (38.7%)	252 (16.3%)	< 0.001
Opportunity risk (4 items)	0 (0.72)	0.67 (0.93)	−0.02 (0.68)	−0.26(0.41)	< 0.001	0.67 (0.99)	−0.14 (0.55)	< 0.001
Alcohol availability at home	0 (1)	0.45 (1.39)	0.06 (1.11)	−0.20 (0.65)	< 0.001	0.49 (1.44)	−0.10 (0.84)	< 0.001
Alcohol availability at social gatherings (3 items)	0 (0.81)	0.88 (1.07)	−0.10 (0.58)	−0.31 (0.41)	< 0.001	0.85 (1.10)	−0.18 (0.60)	< 0.001
Support protection (6 items)	0 (0.55)	−0.09 (0.56)	−0.03 (0.55)	0.05 (0.54)		−0.15 (0.58)	0.03 (0.54)	< 0.001
Parental support (4 items)	0 (0.68)	−0.19 (0.69)	−0.09 (0.67)	0.10 (0.65)	< 0.001	−0.24 (0.68)	0.05 (0.66)	< 0.001
Controls protection								
Individual controls protection (3 items)	0 (0.77)	−0.32 (0.96)	− 0.03 (0.75)	0.13 (0.65)	< 0.001	− 0.42 (0.94)	0.09 (0.68)	< 0.001
Social control protection (9 items)	0 (0.57)	−0.37 (0.60)	−0.04 (0.55)	0.16 (0.49)	< 0.001	−0.47 (0.59)	0.10 (0.51)	< 0.001
Models protection (4 items)	0 (0.62)	−0.19 (0.65)	−0.04 (0.60)	0.09 (0.59)	< 0.001	−0.22 (0.64)	0.04 (0.60)	< 0.001

Hazard drinking = AUDIT score “5+”, Low Risk Drinking is AUDIT scores “1,2,3,4”; Non-drinkers is AUDIT score “0”

*Significance of differences between group analyzed using univariate ordinal multinomial cumulative logit analysis using GEE methods to adjusted for clustering by school

**Significance of differences between group analyzed using univariate binomial logit analysis using GEE methods to adjusted for clustering by school

Risk Factor Measures: standardize scores where the mean = 0 and standard deviation =1, higher scores are worse. Protective Factor Measure: standardize scale where mean = 0, standard deviation = 1, higher scores are better

As a secondary analysis, we investigated the association between extreme risk and protective factors and drug use, and alcohol use defined as 1) AUDIT score of 0 (no alcohol use) vs. AUDIT score of ≥1 (alcohol use), and 2) AUDIT score of 0 to 4 (no alcohol use to lower-risk alcohol use vs. AUDIT score of 5+ (hazardous use). We also examined the relationship between drug use and 1) alcohol use (AUDIT score > 0) and 2) hazardous alcohol use (AUDIT score ≥ 5). Relative risk and 95% confidence intervals (CI) were computed with univariate binominal logit analysis using GEE methods and specifying an independent correlation structure to reflect the clustered data pattern (See Table 3).

**Table 3:** Relative Risks for Alcohol Use, Hazardous Alcohol Use, and Drug Use Among Secondary School Students In Botswana

	Relative Risk for Drinking Alcohol (AUDIT score 0 vs. 1+) (95% CI)	Relative Risk for Hazardous Drinking (AUDIT score 0–4 vs. 5+) (95% CI)	Relative Risk for Drug Use (95% CI)
Demographic factors			
Male vs. female	1.3 (1.1–1.6)**	1.7 (1.3–2.1)***	2.2 (1.6–3.0)***
Urban vs. peri-urban	1.2 (1.0–1.4)*	1.3 (1.0–1.7)*	1.2 (0.9–1.8)
Models Risks			
Sibling drinks alcohol	1.3 (1.2–1.5)***	1.7 (1.4–2.1)***	1.5 (1.2–1.8)***
Problem drinker at home	1.3 (1.2–1.5)***	1.6 (1.4–1.8)***	1.7 (1.4–2.1)***
High peer models risk	1.4 (1.1–1.6)**	1.7 (1.2–2.2) **	1.6 (1.2–2.0)**
Vulnerability risks			
High individual vulnerability risk	1.7 (1.5–1.9)***	2.4 (2.0–2.9)***	3.1 (2.6–3.9)***
High social vulnerability risk	1.4 (1.3–1.6)***	1.9 (1.5–2.5)***	2.4 (1.9–2.9)***
Suicidal ideation (SI) in past year vs. no SI	1.6 (1.4–1.8)***	2.3 (2.0–2.6)***	2.5 (2.2–2.9)***
Opportunity risk			
High opportunity risk	2.1 (1.9–2.2)***	3.5 (2.9–4.4)***	3.5 (2.9–4.1)***
Support protection			
Low support protection	1.2 (1.1–1.4)***	1.5 (1.3–1.8)***	1.7 (1.4–2.1)***
Controls protection			
Low individual controls protection	1.5 (1.4–1.7)***	2.1 (1.6–2.6)***	2.7 (2.1–3.5)***
Low social control protection	2.0 (1.8–2.2)***	3.1 (2.6–3.8)***	4.0 (3.3–4.8)***
Models protection			
Low models protection	1.5 (1.4–1.7)***	1.9 (1.5–2.3)***	1.8 (1.4–2.3)***
Alcohol use			
Alcohol Use vs. No use	–	–	6.9 (5.7–8.5)***
Hazardous Alcohol Use vs. No use	–	–	10.3 (8.3–12.7)***

Composite models risk variable dichotomized, 1 = 1 standard deviation (sd) above the mean z- score or greater, 0 = less than 1 sd above the mean

Composite protective variable dichotomized, 1 = 1 sd below the mean or less, 0 > 1 sd below the mean

*** *p*-value ≤0.001, ** *p*-value ≤0.01, *p-value ≤0.05

### Multivariate analysis

For a comprehensive understanding of alcohol use, we conducted 4 multivariate ordinal-multinomial cumulative logit analysis with GEE methods. Model 1 examined the association between alcohol and all risks factors together. Model 2 added the demographic variables to the model. Model 3 added the composite protective factors to the model; and Model 4 replaced the Social Controls domain composite protective factors with the specific social subcomponent protective measures. We computed the score Chi-square test for the proportional odds assumption that one set of estimates was similar for all group comparisons. When the assumption was violated, we conducted a multivariate nominal-multinomial logit analysis with GEE methods and calculated 2 sets of estimates. One set reflects the hazardous alcohol use vs. no alcohol use comparison, and the other set of estimates reflect the hazardous drinkers vs. lower-risk drinkers comparison (See Table 4).

**Table 4:** Multinomial-ordinal and Multinomial-nominal Models of Factors Predicting Alcohol Use by AUDIT Score Category among Secondary School Students in Botswana

	Model 1A Hazardous alcohol use vs. no use.		Model 1B Hazardous alcohol use vs. lower risk use		Model 2A Hazardous alcohol use vs. No use		Model 2B Hazardous alcohol use vs. lower-risk use		Model 3		Model 4	
	OR	95%CI	OR	95%CI	OR	95%CI	OR	95%CI	OR	95%CI	OR	95%CI
Models Risk												
Peer models risk	1.55**	1.13–2.13	1.18	0.92–1.51	1.63**	1.11–2.27	1.14	0.90–1.44	1.32**	1.09–1.59	1.28**	1.06–1.54
Sibling drinks alcohol	1.23	0.84–1.79	1.28	0.90–1.81	1.29	0.88–1.89	1.32	0.94–1.85	1.21	0.94–1.57	1.20	0.93–1.56
Problem drinker at home	0.95	0.65–1.39	1.18	0.84–1.66	1.01	0.70–1.47	1.21	0.86–1.71	1.12	0.91–1.38	1.11	0.90–1.38
Opportunity Risk												
Alcohol availability at home	1.42***	1.21–1.67	1.08	0.91–1.28	1.38***	1.19–1.60	1.07	0.90–1.27	1.29***	1.16–1.42	1.29***	1.17–1.43
Alcohol availability in community	7.26***	5.26–10.0	4.01***	2.87–5.59	7.21***	5.32–9.77	3.85***	2.72–5.46	4.19***	3.51–5.00	4.02***	3.38–4.78
Vulnerability Risk												
Social Vulnerability Risk	1.03	0.84–1.25	1.06	0.82–1.37	1.08	0.90–1.31	1.08	0.84–1.39	1.10	0.92–1.31	1.10	0.93–1.30
Individual Vulnerability Risk												
Low perception of self	0.91	0.67–1.24	0.73	0.52–1.03	0.93	0.70–1.24	0.72	0.51–1.03	1.05	0.82–1.34	1.10	0.85–1.41
Low expectations of the future	1.76***	1.43–2.17	1.63***	1.28–2.08	1.75***	1.40–2.18	1.55***	1.19–2.02	1.22**	1.05–1.43	1.23**	1.05–1.44
Suicidal Ideation	1.28	0.92–1.80	1.18	0.96–1.45	1.40*	1.00–1.95	1.28	0.97–1.68	1.35**	1.07–1.70	1.29*	1.02–1.63
Demographic variables												
Age					0.89*	0.79–1.00	1.05	0.94–1.18	0.90*	0.83–0.98	0.90	0.82–0.98
Male gender					1.66**	1.11–2.48	1.43	0.94–2.19	1.24	0.92–1.66	1.25	0.93–1.66
Urban location					1.41	0.98–2.04	1.29	0.91–1.83	1.12	0.86–1.45	1.13	0.87–1.46
Support Protection									1.20	0.94–1.53	1.20	0.95–1.53
Models Protection									0.90	0.73–1.11	0.90	0.73–1.11
Controls Protection												
Individual Controls Protection									0.85	0.70–1.02	0.84	0.70–1.01
Social Controls Protection									0.66**	0.49–0.88		
Family monitoring											0.77**	0.65–0.91
Peer disapproval of substance use											0.79***	0.69–0.90
Peers view academic achievement as important											1.02	0.89–1.16
School Attachment											1.00	0.90–1.13

*** p-value ≤0.001, ** p-value ≤0.01, *p-value ≤0.05

Similarly for drug use (yes vs. no), we conducted 4 multivariate binominal logit analysis using GEE methods that adjust for clustered data. Model 1 examined the association between drug use and all risks factors together. Model 2 added the demographic variables to the model. Model 3 added the composite protective factors to the model; and Model 4 replaced the social domain composite protective factors with the specific social subcomponent protective measures (See Table 5).

**Table 5:** Binomial logit models of risk and protective factors predicting drug use among secondary school students in Botswana

	Model 1		Model 2		Model 3		Model 4	
	OR	95% CI	OR	95% CI	OR	95% CI	OR	95% CI
Models Risk								
Peer models risk	1.06	0.86–1.31	1.06	0.86–1.30	1.01	0.81–1.26	0.94	0.75–1.19
Sibling drinks alcohol	1.01	0.76–1.34	1.00	0.75–1.33	1.09	0.85–1.41	1.12	0.87–1.44
Problem drinker at home	1.11	0.80–1.54	1.12	0.82–1.52	1.19	0.88–1.62	1.21	0.87–1.67
Opportunity Risk								
Alcohol availability at home	1.16*	1.02–1.33	1.14*	1.01–1.29	1.09	0.98–1.22	1.09	0.97–1.22
Alcohol availability in community	2.76***	2.26—3.36	2.56***	2.10–3.14	2.22***	1.79–2.76	2.11***	1.71–2.62
Vulnerability Risk								
Social Vulnerability Risk	1.14	0.91–1.94	1.22	0.97–1.52	1.27*	1.02–1.58	1.28*	1.02–1.60
Individual Vulnerability Risk								
Low perception of self	1.38	0.98–1.94	1.39	0.99–1.96	1.15	0.84–1.58	1.21	0.87–1.67
Low expectations of the future	1.44**	1.09–1.92	1.36*	1.03–1.79	1.18	0.88–1.59	1.20	0.88–1.62
Suicidal Ideation	1.40**	1.09–1.80	1.73***	1.28–2.32	1.65**	1.16–2.35	1.61**	1.11–2.33
Demographic variables								
Age			1.03	0.90–1.17	1.04	0.91–1.18	1.02	0.89–1.17
Male gender			2.51***	1.70–3.70	2.10***	1.43–3.08	2.11***	1.47–3.01
Urban location			1.28	0.90–1.83	1.23	0.88–1.70	1.18	0.86–1.63
Support Protection					1.14	0.83–1.55	1.10	0.79–1.52
Models Protection					1.18	0.90–1.55	1.22	0.93–1.60
Controls Protection								
Individual Controls Protection					0.82	0.65–1.02	0.80*	0.64–1.00
Social Controls Protection					0.36***	0.26–0.51		
Family monitoring							0.77	0.59–1.02
Peer disapproval of substance use							0.57***	0.49–0.65
Peers view academic achievement as important							1.03	0.92–1.15
School attachment							0.77*	0.59–1.00

*** p-value ≤0.001, ** p-value ≤0.01, *p-value ≤0.05

### Study size

Using data from the 2005 GSHS Botswana survey on drug and alcohol use and risk factors for alcohol and drug use, estimating an average 3:1 ratio between students not exposed to risk to those exposed to risk [1], we calculated that a sample size of 1364 was needed to reject the null hypothesis—that the occurrence of alcohol and drug use among secondary school students exposed to certain risk factors is equal to the occurrence among those unexposed to the same risk factor—with a power of 80% and type I error probability of 0.05. We based class recruitment on estimates of 35 students/class for public senior secondary schools, 40 for junior secondary schools, and 30 for private secondary schools, and anticipated inviting approximately 1990 students to participate in the study to ensure that the sample size would be adequate.

## Results

### Design effect and effective sample size

Given the cluster sample design, the calculated intra-cluster correlation coefficient (ICC) was 0.02, rendering a design effect of 3.26, and yielding an effective sample size of *n* = 594.

### Demographic characteristics (See Table 1)

All 1936 students present in class on the day of the survey opted to participate in the study. Of these participants, 1029 (53.2%) were females and 904 (46.7%) were male, and 3 (0.1%) did not specify their gender. The mean age was 16 years with an interquartile range of 14 to 17 years. There were 1173 (60.6%) participants from urban schools (Gaborone), and 763 (39.4%) went to school in a peri-urban environment (Molepolole, Lobatse, and Mochudi). There were 402 (20.8%) Form 1 students, 371 (19.2%) Form 2 students, 360 (18.6%) Form 3 students, 566 (29.2%) Form 4 students, and 237 (12.2%) Form 5 students included in the study. Table 1 describes demographic variables and group differences by AUDIT score categories and illicit drug use.

### Associations between risk and protective factors and substance use

While some students left survey questions unanswered, unanswered questions accounted for less than 2.6% of responses per item. Of the 1936 students surveyed, 1927 (99.5%) students completed the AUDIT questions, 816 (42.1%) students reported alcohol use, and 434 (22.5%) met the threshold for hazardous alcohol use. A total of 1885 (97.4%) students answered survey questions on drug use, 324 (16.7%) reported drug use, with 155 (8.0%) students reporting motokwane (marijuana) as the drug they used most often. Relative risks for adolescent alcohol use, hazardous drinking, and drug use were calculated in regard to demographic information as well as to a number of risk and protective factors and are presented in Table 3. While males were found to be 1.3 (95% CI: 1.1, 1.6) times more likely to drink alcohol than females (48.4% vs. 36.8%, *p* ≤ 0.01), they were 1.7 (95% CI: 1.3, 2.1) times more likely to drink hazardously (28.4% vs. 17.1%, *p* ≤ 0.001). Males were also 2.2 (95%: 1.6, 3.0) times more likely to use drugs (25.3% vs 14.5%, *p* ≤ 0.001).

Each risk domain (models risk, individual vulnerability risk, social vulnerability risk, and opportunity risk) was modeled separately for relation to reported behavior (See Table 3). While all risk groups were positively associated with reported alcohol and drug use behaviors, the highest relative risk was associated with the factors highlighted below. Adolescents at high opportunity risk were 2.1 (95% CI: 1.9, 2.3) times more likely to drink alcohol (74.8% vs. 36.2%, *p* ≤ 0.001), 3.5 (95% CI: 2.9, 4.4) times more likely to hazardously drink (56.9% vs. 16.0%, *p* ≤ 0.001), and 3.5 (95% CI: 2.9, 4.2) times more likely to use drugs compared to those without high opportunity risk (43.1% vs. 12.3%, *p* ≤ 0.001). Adolescents at high individual vulnerability risk were 1.7 (95% CI: 1.5, 1.9) times more likely to drink alcohol (63.8% vs. 37.9%, *p* ≤ 0.001), 2.4 (95% CI: 2.0, 2.9) times more likely to hazardously drink (43.7% vs. 18.1%, *p* ≤ 0.001), and 3.1 (95% CI: 2.6, 3.9) times more likely to use drugs (39.3% vs. 12.5%, *p* ≤ 0.001).

With regard to protective factors, particularly notable are the effects of low individual controls protection and low social controls protection. Adolescents with low individual controls protection were 1.5 (95% CI: 1.3, 1.8) times more likely to drink alcohol (60.9% vs. 39.6%, *p* ≤ 0.001), 2.1 (95% CI: 1.6, 2.6) times more likely to hazardously drink (40.7% vs. 19.8%, *p* ≤ 0.001), and 2.7 (95% CI: 2.1, 3.5) times more likely to use drugs (38.5% vs. 14.1%, p ≤ 0.001). Adolescents with low social controls protection were 2.0 (95% CI: 1.8, 2.2) times more likely to drink alcohol (73.2% vs. 36.4%,* p* ≤ 0.001), 3.1 (95% CI: 2.6, 3.8) times more likely to drink hazardously (52.4% vs. 16.7%, *p* ≤ 0.001), and 4.0 (95% CI: 3.3, 4.8) times more likely to use drugs (46.1% vs. 11.6%, *p* ≤ 0.001). Lastly, adolescents were 10.3 (95% CI: 8.2, 12.7) times more likely to use drugs if they use alcohol hazardously as compared to no alcohol use (50.1% vs. 4.9%, *p* ≤ 0.001).

Particularly surprising to us among the results of survey data was participant responses to whether or not they “seriously considered attempting suicide in the past year.” 381 students (19.7%) answered “yes.” Of note, 36.0% of the Form 5 female students and 31.9% of Form 4 females endorsed seriously considering suicide in the past year compared to only 17.3% of male Form 5 and 14.6% of Form 4 male students. Alcohol and drug use risks were calculated in regard to the presence of suicidal ideation and it was found that adolescents reporting suicidal ideation were 1.6 (95% CI: 1.4, 1.8) times more likely to drink alcohol (59.6% vs. 37.5%, *p* ≤ 0.001), 2.3 (95% CI: 2.0, 2.6) times more likely to hazardously drink (39.9% vs. 17.7%, *p* ≤ 0.001), and 2.5 (95% CI: 2.2, 2.9) times more likely to use drugs (31.9% vs. 12.6%, *p* ≤ 0.001).

### Multivariable models

#### Alcohol use

Multinomial regression models, controlling for socio-demographic information and adjusted for clustering by school were created to better understand the relationship between alcohol and drug use and risk and protective factors. Model 1A for hazardous alcohol use vs. no use identified models risk (OR 1.55, *p* ≤ 0.01), alcohol availability at home (OR 1.42, *p* ≤ 0.001), alcohol availability in the community (OR 7.26, *p* ≤ 0.001) and low future expectations (OR 1.76, *p* ≤ 0.001) as predictive of hazardous alcohol use. Model 1B for hazardous use vs. lower-risk alcohol use, alcohol availability in the community (OR 4.01, *p* ≤ 0.001) and low expectations for the future were predictive of hazardous alcohol use. In both Model 2A and 2B, which control for age, gender, and school location, the same risk and protective factors remain predictive of hazardous alcohol use. Model 3 adds protective factors including support protection, models protection, individual controls protection and social control protection. Models risk and opportunity risk remain predictive in Model 3 after adding in protective factors, however the odds ratio for alcohol availability in the community decreases to 4.19 (*p* ≤ 0.001). Social controls protections are significantly protective in Model 3 (OR 0.66, *p* ≤ 0.01). Model 4 controls for demographic factors, and includes subcategories of individual vulnerability risk factors and social controls protections. In this final model, predictive factors include models risk (OR 1.28, *p* ≤ 0.01), opportunity risks including alcohol availability at home (OR 1.29, *p* ≤ 0.001) and alcohol availability in the community (OR 4.02, *p* ≤ 0.001), low future expectations (OR 1.23, p ≤ 0.01), suicidal ideation in the past year (OR 1.29, *p* ≤ 0.05), family monitoring (OR 0.77, *p* ≤ 0.01), and peer controls protection related to disapproval of substance use (OR 0.79, *p* ≤ 0.001).

#### Drug use

Model 1 looking at student drug use identified opportunity risks including alcohol availability at home (OR 1.16, *p* ≤ 0.05), alcohol availability in the community (OR 2.76, *p* ≤ 0.001), individual vulnerability risks including low expectations of the future (OR 1.44, *p* ≤ 0.001), and suicidal ideation within the past 12 months (OR 1.40, *p* ≤ 0.01) as predictive factors. When age, male gender, and school location (urban vs. peri-urban) were controlled for in Model 2, the same opportunity risks and individual vulnerability risks remained predictive with similar odds ratios. Model 3, which incorporates protective factors, includes the following predictive factors: alcohol availability in the community (OR 2.22 *p* ≤ 0.001), social vulnerability risk (OR 1.27, *p* ≤ 0.05), suicidal ideation (OR 1.65, *p* ≤ 0.01), and social controls protection (OR 0.36, *p* ≤ 0.001). The final model shows the opportunity risk of alcohol availability in the community (OR 2.11, *p* ≤ 0.001), social vulnerability risk (OR 1.28, *p* ≤ 0.05), seriously considering suicide in the past year (OR 1.61, *p* ≤ 0.01) all significantly predict student drug use. Individual controls protection (OR 0.80, *p* ≤ 0.05), and social controls protection including peer disapproval of alcohol or illicit drug use (OR 0.57, *p* ≤ 0.001), and school attachment (OR 0.77, *p* ≤ 0.05) are protective against drug use in Model 4.

## Discussion

This study investigated risk and protective factors that influence the prevalence of alcohol and drug use among secondary school students in Botswana. These results can aid the development of policies and programming designed to reduce adolescents’ high consumption of alcohol and drugs in this and similar settings. Our data suggest a number of practical solutions to target this issue.

The prevalence rates of student alcohol and drug use found by our study is significantly higher than previously reported rates in Botswana, lending quantitative support to the concerns raised by local educational professionals. Our study found that 42.1% of secondary school students reported alcohol use (defined as an AUDIT score > 0), more than twice the rate (18.9%) found among of upper primary and secondary students surveyed during the 2010 Botswana Ministry of Education (MOE) Youth Risk Behavior Surveillance Report, and the 20.5% of secondary students ages 13–15 who reported alcohol use in the past 30 days in the 2005 WHO Global School-based Student Health Survey [1, 2]. Rates of hazardous drinking also appear to be higher: Our study showed 22.5% of secondary school students had AUDIT scores indicative of hazardous drinking compared to 16.6% Batswana students ages 13–15 reporting adverse consequences from drinking alcohol in the WHO 2005 Global School-based Student Health Survey. The rate of ever having used illicit drugs (16.7%) was also higher than seen in previously reported studies [1, 2]. In the 2010 Youth Behavior Surveillance Survey Report conducted by the Botswana MOE, 13.2% of students reported marijuana use one or more times. It is unclear if the higher prevalence rate of student substance use seen in our study is due to temporal changes or geographical differences.

While little prior data existed to explain risk factors for substance use among youth in Botswana, studies from neighboring South Africa offer opportunities for comparison. A study conducted in rural South Africa, identified several community influences associated with adolescent use of home-brewed alcohol. Positive correlations existed between alcohol use and the number of adults an adolescent knew to “engage in antisocial behaviors,” such as drug use, as well as between alcohol use and an adolescent’s perception of his or her neighborhood being in a “derelict state and being unsafe.” On the other hand, a negative correlation existed between alcohol use and perceived community affirmation [26]. It has also been shown that there is a correlation between physical punishment during childhood and alcohol use later in life [27].

Within peer-reviewed literature, our findings are consistent with several other studies demonstrating the high prevalence of substance use in Botswana and Southern Africa, including among young adults and college students [28–31]. This study broadens the evidence base by specifically investigating a younger school-going adolescent population. In our study, 35.1% of Form 1 students and 40.4% of Form 2 students (equivalent to grades 8 and 9 in American schools) reported alcohol use. This finding highlights the importance of alcohol and drug use prevention interventions that target younger adolescents.

Our data suggest a number of practical solutions to target adolescents’ high consumption of alcohol and drugs in this and similar settings. Prominent risk factors for alcohol and drug use among secondary school students include opportunity risk, models risk, and vulnerability risk. Most remarkably, adolescents with easy access to alcohol, whether at home or in their community, were 3.5 times more likely to drink hazardously or use drugs (see Table 3). This finding is consistent with studies examining adult populations, which likewise found that easy availability of alcohol, including home-brewed alcohol, and its use in gatherings and community activities, contributes to alcohol abuse in Botswana [31–33]. Interventions aimed at reducing the availability of alcohol and drugs for secondary school students could include increased funding for enforcement of underage drinking laws to ensure that businesses or individuals are not selling alcohol to minors. A similar intervention had a mitigating effect on adolescent drinking in California, USA [34]. Alternatively, regulating the density of places where alcohol is both purchased and consumed may decrease alcohol availability, and thus adolescent substance use [35]. The prevalence of alcohol and drugs in students’ homes and in social gatherings would be more difficult to control, as alcohol use is a deeply embedded social norm in Botswana [33]. However, programming could be developed for parents to educate them about the risks of having alcohol available at home and ways to reduce their children’s access. While several studies positively correlate parental monitoring, disapproval of adolescent drinking, parental modeling, parent-child relationship quality, and general communication with decreased adolescent alcohol use, future studies are needed to evaluate the effectiveness of parental interventions on adolescent substance use [36–38]. For example, a recent systematic review and meta-analysis on the effects of parental alcohol rules on risky drinking pooled evidence from 13 studies conducted in the US, Sweden and the Netherlands and found that teens with parents who set rules concerning alcohol were less likely to develop risky drinking or related problems (OR = 0.64, 95% CI: 0.48, 0.86) [38]. Further studies within the Southern African context are needed to determine if interventions aimed at supporting and changing alcohol and illicit drug-related parenting are similarly protective.

Adolescents who report having family members or peers who use drugs or alcohol are considered to have models risks. Our study found that adolescents at high peer models risk were 1.7 times more likely to drink hazardously and 1.6 times more likely to use drugs. This finding concurs with studies of adolescent populations all around the world [39–44]. A recent study in neighboring Zimbabwe echoed the positive correlation between family members and friends who use cannabis and student cannabis use [45]. Programming intended for the parents of adolescents with models risks may be beneficial for the entire family. Programming would also need to target adolescents themselves, as adolescents model the behaviors of their peers; for example, students could to be taught skills to resist peer pressure or be provided with more extracurricular activities. Though the evidence base is limited, a recent review of 17 studies indicates that peer-led interventions to decrease youth substance use may be effective, and may be a way to harness greater youth engagement [46]. Further research should identify the school-based interventions that adolescents themselves believe would be most successful. Some studies conducted in the US and Europe found that school-based interventions, which focus on general psychosocial development and life skills, as opposed to interventions that aim only to increase knowledge and awareness about substances, may be effective in reducing alcohol use as well as other problematic behaviors [47–49].

Our multivariable models show that individual and social controls protection contributes to a decreased risk of alcohol and drug use (see Tables 4 and 5), although they do not completely eliminate risk. These protective factors, which include peer disapproval of substance use, school attachment, family monitoring, and religiosity, most likely help individuals develop core beliefs that protect against substance use, as well as the coping strategies they need to handle the stresses they feel at home, in their social lives, or at school. For example, individuals who reported school attachment may cope with problems by discussing them with their teachers or friends, rather than by drinking alcohol or using drugs. These findings may be helpful for designing interventions. For example, drug and alcohol programming that targets parents could also educate them about the importance of supporting their children’s success at school and being available and willing to openly discuss their children’s problems. Improvements in the counseling services provided at school might also help students learn how to better cope with these stresses.

Not surprisingly, both alcohol and drug use are more prevalent among male adolescents compared to female adolescents in our study. This is concordant with studies of older individuals in Botswana [31, 32]. Although not the focus of this study, the high rates of suicidal ideation among older female adolescents presented a concerning glimpse at a different type of risk in the female students. While the overall prevalence of suicidal ideation (19.7%) found in our study is similar to that reported in other low to mid-income countries, the prevalence of suicidal ideation among female senior secondary students was particularly high [50–53]. Our study found that 31.9% of Form 4 girls and 36.0% of the Form 5 female students endorsed serious suicidal ideation within the past 12 months, and that suicidal ideation was correlated with significant increases in drug and alcohol use. Risk and protective factors for emotional dysregulation, poor distress tolerance, depression and suicidality need to be further investigated among secondary school students in Botswana, particularly among older school-going girls.

There are a number of limitations to the results of this study that are important to recognize. First, because the study is cross-sectional, we are not able to make causal judgments based on our findings. Second, all data are self-reported in the form of surveys completed at school. While students were ensured anonymity, self-reported data presents the possibility of bias in that students may have answered what they deemed was socially desirable [54, 55]. Third, there are a number of other biological, social and environmental factors we did not measure through our survey which may have effects on student drinking and drug use, such as genetic predisposition to alcohol abuse, socioeconomic status, trauma, bullying, community disorganization, poor schooling and teacher absenteeism [11]. Our study also neglected to examine adolescent tobacco use, which is a prevalent risky behavior among secondary school students in Botswana [56].

There may also be some limitations to the generalizability of our findings. Only students of Gaborone and its peri-urban locations were surveyed. While we believe that these students are similar in many ways to those in other parts of Botswana and throughout Southern Africa, the extent to which the findings are applicable to students living in rural areas is unknown. There were in fact slight differences in how students from Gaborone versus peri-urban areas answered some questions. Substance use may also be more of a social norm in urban areas, perhaps explaining greater substance use among urban adolescents [31].

While few questions were left unanswered, internal consistency was low for questions related to a few of the constructs. In particular, the Cronbach’s alpha for the models risk construct, which included four survey questions, was 0.28. This suggests that individual items within the construct do not commonly co-exist with other items within the construct.

## Conclusion

This study increases our understanding of substance use among school-going adolescents in Botswana. The ease with which adolescents can obtain alcohol and drugs greatly contributes to their use. In addition, adolescents who experienced stresses in family and peer relationships or stresses at school were at risk for alcohol and drug use, but those who had support from friends and family, school attachment, goals for the future, and religiosity were more protected from the risks of substance use than those who did not. Interventions should therefore reduce the availability of alcohol and drugs and attempt to build greater support networks for adolescents who do not have them. The high rate of suicidal ideation among secondary school students in Botswana also indicates a pressing need for mental health prevention and promotion interventions within schools.

## Additional file

Additional file 1: **Table S1.** Survey Risk and Protective Factor Items and Response Codes. **Table S2.** Factor Analysis Findings for Problem Behavior Theory Risk Factor Survey Items. **Table S3.** Factor Analysis Findings for Problem Behavior Theory Protective Factor Survey Items. (DOCX 48 kb)

## Abbreviations

AUDIT: Alcohol Use Disorder Identification Test
CI: Confidence intervals
GEE: Generalized Estimating Equations
GSHS: Global School-based Health Survey
ICC: Intracluster correlation coefficient
ID: Identification
IRB: Institutional Review Board
MOE: Ministry of Education and Skills Development
MSA: Kaiser’s Measure of Sampling Adequacy
PBT: Problem Behavior Theory
SD: Standard deviation
WHO: World Health Organization
