# Supplementary material for: Prevalence and predictors of alcohol and drug use among secondary school students in Botswana: a cross-sectional study
Source: BMC Public Health. 2018 Dec 20;18:1396. doi: 10.1186/s12889-018-6263-2 (PMC6302490; doi:10.1186/s12889-018-6263-2)
Supplement: Supplementary file 1 — Table S1. Survey Risk and Protective Factor Items and Response Codes. Table S2. Factor Analysis Findings for Problem Behavior Theory Risk Factor Survey Items. Table S3. Factor Analysis Findings for Problem Behavior Theory Protective Factor Survey Items. (DOCX 48 kb) [file 12889_2018_6263_MOESM1_ESM.docx]

Table S1 Survey Risk and Protective Factor Items and Response Codes

| **Risk and Protective Factors Assessment** | | |
| --- | --- | --- |
|  | Items | Response Codes |
|  | ***Social Controls Protection*** |  |
| 4 - 6 | *Family Monitoring*   - How much would you say your parent(s)/guardian(s) really know what you do during your free time? - How much would you say your parent(s)/guardian(s) really know how you spend your money? - How much would you say your parent(s)/guardian(s) really know where you spend time on weekends? | 1 (Never know) to 3 (Always know) |
| 7 | - How often does your parent/guardian scold or reprimand you when you do something wrong; for example, if you come home late, don’t do your chores, watch too much tv? *(dropped from analysis due to frequent student confusion regarding question during implementation)* | 1 (Never) to 5 (Every time) |
| 8 | *Peer control values*   - How important is it to your friends that you do well in school? | 1 (Not important) to 3 (Very important) |
| 9 - 10 | - How do most of your friends feel about someone your age drinking alcohol? - How do most of your friends feel about someone your age using marijuana or other drugs? | 1 (Strongly approve) to 4 (Strongly disapprove) |
| 11 - 13 | *School attachment*   - In general, I like school a lot. - In general, I get along well with my teachers. - I try my best at school. | 1 (Strongly disagree) to 5 (Strongly agree) |
|  | ***Individual Controls Protection*** |  |
| 14 - 16 | *Religiosity*   - How important is it to you to believe in God? - How important is it to you to rely on religious beliefs as a guide for day-to-day living? - How important is it to be able to prayer when you are facing a personal problem? | 1 (Not important) to 3 (Very important) |
|  | ***Models Protection*** |  |
| 17 - 20 | *Positive Peer Modeling*   - How many of your friends get good marks in school? - How many of your friends participate in sports? - How many of your friends attend church/mosque/temple? - How many of your friends want to go to university or college? | 1 (None of them) to 4 (All of them) |
|  | ***Support Protection*** |  |
| 21 - 24 | *Parental closeness*   - Since the beginning of this school year, how often have you talked to a parent or guardian about problems you were having at school? - How often does your parent/guardian teach you things you didn’t know? - How often do you share secrets or private feelings with your parent/guardian? - How often does your parent/guardian try to help you when you need something? | 1 (Never) to 5 (Always) |
| 25 | *Teacher Closeness*   - The teachers at my school will spend extra time to help pupils/students do their best. | 1 (Strongly disagree) to 4 (Strongly agree) |
| 26 | *Peer Closeness*   - How many close friends do you have? | 1 (0) to 4 (3 or more) |
|  | ***Models Risk*** |  |
| 27 - 28 | *Poor family modeling*   - Have any of your brothers or sisters ever drunk alcohol or currently drink alcohol? - Have you ever lived with anyone who was a problem drinker or alcoholic? | 1 (Yes) or 0 (No) |
| 29 | *Poor peer modeling*   - Drinking and drug use is a problem for students at my school. | 1 (Strongly disagree) to 5 (Strongly agree) |
| 30 | - How many of your friends drink alcohol? | 1 (None of them) to 4 (All of them) |
|  | ***Opportunity Risk*** |  |
| 31 | *Alcohol availability*   - If you wanted to drink alcohol, would you be able to get it at home? | 1 (Never) to 5 (Always) |
| 32 - 34 | - How many times have you lied about your age to buy alcohol? - How many times has someone bought alcohol for you? - How many times has someone offered you alcohol at a party or wedding? | 1 (Never) to 5 (6 or more times) |
|  | ***Social Vulnerability Risk*** |  |
| 35 | *Conflict at home*   - Is there tension or stress at home in your family? | 1 (Never) to 5 (Always) |
| 36 | *Conflict in social life*   - In the past 6 months, how much stress or pressure have you felt in your personal or social life? | 1 (None) to 4 (A lot) |
| 37 | *Conflict at school*   - In the past 6 months, how much stress or pressure have you felt at school? | 1 (None) to 4 (A lot) |
|  | ***Individual Vulnerability Risk*** |  |
| 38 - 40 | *Low perception of self*   - How well do you get along with others your age? - How well do you live up to what other people expect of you? - What about your ability to do well in school? | 1 (Very well) to 4 (Not very well at all) |
| 41 | - On the whole, how satisfied are you with yourself? | 1 (Very satisfied) to 4 (Not satisfied at all) |
| 42 - 45 | *Low expectations for the future*   - What are the chances that you finish secondary school? - What are the chances that you will be able to go to university? - What are the chances that you will have a job that pays well? - What are the chances you will be respected in your community? | 1 (High) to 3 (Low) |
| 46 | *Depressive symptoms*   - During the past 12 months, did you ever seriously consider attempting suicide? | 1 (Yes) or 0 (No) |

Table S2 Factor Analysis Findings for Problem Behavior Theory Risk Factor Survey Items

| **PBT (42 items)** | Theory blueprint | [a]  **>0.60** standardized  coefficient alpha | Inter-Item Correlation | Partial Correlation | [b]  MSA Item | [b]  **> 0.50**  MSA  Overall | [c]  **>1.0**  Factor  Eigen  value | [d]  % variance | [e]  **> 0.70**  **min > 0.60** Factor Loadings | SMC Orig | [f]  **>48%,** **min >36%** Final % commonality | Scoring Of Risk Factors in Each Domain |
| --- | --- | --- | --- | --- | --- | --- | --- | --- | --- | --- | --- | --- |
| **RISK FACTORS (19 items)** |  |  |  |  |  |  |  |  |  |  |  |  |
| **Domain1**: **Models Risk**  Sibling drink q27  Problem drinker at home q28  High peer model risks | q27:YN, q28YN,  q29, q30(Likert 1 to 4pts) | 0.26, 0.32 | 0.08 - 0.17 | 0.06 - 0.15 | 0.55 - 0.62 | 0.57 | 1.32 | 33% | 0.41 to 0.69 | 1.0 | 0.17 to 0.48 | Xcomposite_Risk_D1_RoleModels =   mean(zq29,zq30); |
| **Domain2**:**Opportunity Risk** | q31, q32, q33, q34 (Likert 1 to 5pts) | 0.70 | 0.22 - 0.64 | 0.08 - 0.56 | 0.62 - 0.83 | 0.67 | 2.11 | 53% | 0.47, 0.85, 0.81, 0.71 | 1.0 | 0.22 to 0.72 | XComposite_Risk_D2_Opptunity =  mean(XSub_Risk_D2_Opptunity, zq31); |
| Alcohol availability in community | q32, q33, q34 (Likert 1 to 5pts) | 0.74 | 0.36 - 0.64 | 0.10 - 0.57 | 0.59 - 0.75 | 0.63 | 1.98 | 66% | 0.71, 0.84, 0.88 | 1.0 | 0.51 to 0.77 | XSub_Risk_D2_Opptunity =  mean(zq32,zq33, zq34); |
| **Domain3**:**Vulnerability Risk** | 12items; |  |  |  |  |  |  |  |  |  |  |  |
|  | 3 social vulnerability risk items  q35 (Likert 1 to 5pts)  q36, q37 (Likert 1 to 4pts) | 0.66 | 0.25 - 0.49 | 0.04 - 0.44 | 0.56 - 0.63 | 0.59 | 1.80 | 60% | 0.86, 0.75, 0.72 | 1.0 | 0.51 to 0.74 | XSub_Risk_D3_VlnerblitySocStress =  mean(zq35,zq36, zq37); |
|  | 4 low self-perception,  q38, q39, q40, q41  (1 to 4 Likert scale) | 0.55 | 0.17 - 0.30 | 0.08 - 0.24 | 0.66 - 0.69 | 0.67 | 1.71 | 43% | 0.59, 0.66, 0.67, 0.68 | 1.0 | 0.35 to 0.47 | XSub_Risk_D3_VlnerblitySlfEsteem =  mean(zq38,zq39, zq40, zq41); |
|  | 4 low expectations of the future:  q42, q43, q44, q45 (1 to 3 Likert scale) | 0.70 | 0.23 - 0.60 | 0.05 - 0.52 | 0.63 - 0.77 | 0.67 | 2.12 | 53% | 0.61, 0.82, 0.82, 0.63 | 1.0 | 0.37 to 0.67 | XSub_Risk_D3_VlnerblityFuture =   mean(zq42,zq43, zq44, zq45); |
|  |  |  |  |  |  |  |  |  |  |  |  | Xcomposite_Risk_D3_Vlnerblity =  mean(XSub_risk_D3_VlnerblitySocStress  XSub_risk_D3_VlnerblitySlfEsteem,  XSub_risk_D3_VlnerblityFuture), |
|  | Suicidal ideation in past 12mo (y/n) | n/a | n/a | n/a | n/a | n/a | n/a | n/a | n/a | n/a | n/a | Q46, not added in because Y/N |

[a] Standardize Coefficient Alpha (criterion>0.60), [b] MSA=Kaiser Measure of Sampling Adequacy (Criterion: >5) difference between original correlations & partial correlations, [c] Eigenvalue (criterion > 1.0), [d] Percent of variance explained by a Factor (criterion:> 0.49, min = 0.36), [e] Factor loading: correlations between item & factor (criterion: > 0.70, min 0.60), [f] Final Commonality: % of variance in item explained by Factor, (i.e., factor loading)

Table S3 Factor Analysis Findings for Problem Behavior Theory Protective Factor Survey Items

| **PBT** (n=42 items) | Theory blueprint | [a]  **>0.60** standardize coefficient alpha | Inter-Item Correlation | Partial Correlation | [b]  MSA Item | [b]  **> 0.50** MSA Overall | [c]  **>1.0**  Factor  Eigen value | [d]  % var | [e]  **> 0.70**  **min >0.60** Factor Loadings | SMC Orig | [f]  **>48%**  **min >36%** Final % commonality | Scoring Of Protective Factors in Each Domain |
| --- | --- | --- | --- | --- | --- | --- | --- | --- | --- | --- | --- | --- |
| **PROTECTIVE FACTORS (22 items)** |  |  |  |  |  |  |  |  |  |  |  |  |
| **Domain 1: Models Protection** | 4, 4pt Likert items q17, q18, q19, q20 | 0.46 | 0.07 - 0.27 | 0.02 - 0.22 | 0.62, 0.68, 0.62, 0.64 | 0.63 | 1.55 | 39% | 0.70, 0.41, 0.70, 0.63 | 1.0 | 0.50, 0.17, 0.49, 0.40 | Xcomposite_Protect_D1_PeerModels =  mean(zq17, zq18, zq19, zq20); |
| **Domain2: Control**  **Protection** |  |  |  |  |  |  |  |  |  |  |  |  |
| Parental control | 3, 3pt Likert items q4, q5, q6 | 0.60 | 0.29, 0.33, 0.38 | 0.19 - 0.32 | 0.61, 0.62, 0.67 | 0.63 | 1.67 | 56% | 0.78, 0.75, 0.71 | 1.0 | 0.60, 0.56, 0.50 | XSub_Protect_D2_ControlParent =  mean(zq4, zq5, zq6); |
| Peer Control1 | q8 (3pt Likert)  q9, 10 (4pt Likert) | 0.65 | 0.24,0.25, 0.65 | 0.11, 0.13, 0.63 | 0.82, 0.54, 0.54 | 0.56 | 1.80 | 60% | 0.87, 0.87, 0.53 | 1.0 | 0.28, 0.76, 0.76 | XSub_Protect_D2_ControlPeerchk = mean(XSub_Protect_D2_ControlPeer,zq8) |
| Peer disapproval of substance use | 2,4pt Likert Items  q9, q10 | 0.79 | 0.65 | 0.65 | 0.50, 0.50 | 0.50 | 1.65 | 83% | 0.91, 0.91 | 1.0 | 0.83, 0.83 | XSub_Protect_D2_ControlPeer =  mean(zq9, zq10); |
| School Attachment | 3, 5pt Likert items q11, q12, q13 | 0.61 | 0.31, 0.34, 0.38 | 0.21, 0.25, 0.30 | 0.62, 0.64, 0.66 | 0.64 | 1.68 | 56% | 0.77, 0.75, 0.72 | 1.0 | 0.59, 0.57, 0.52 | XSub_Protect_D2_ControlSchAttach = mean(zq11, zq12, zq13) |
| Social Level Control  -family monitoring  -school attachment  -peer control | q4, q5, q6   q8, q9, q10   q11, q12, q13 | 0.72 | 0.01 - 0.60 | 0.01 - 0.60 | 0.65 to 0.88 | 0.74 | 1.76 1.73 1.71 | 19% 20% 19% | F3: 0.77, 0.75, 0.65 F1: 0.43, 0.85, 0.88 F2: 0.73, 0.74, 0.69 | 1.0 | 0.61, 0.58, 0.46 0.30, 0.78, 0.80, 0.56, 0.58, 0.52 | XComposit_Protect_D2_ControlSoc =  mean(XSub_Protect_D2_ControlParent, XSub_Protect_D2_ControlPeer, XSub_Protect_D2_ControlSchAttach,zq8); |
| Individual Control (belief in God, religion, &prayer) | 3, 3pt Likert items q14, q15, q16 | 0.66 | 0.45, 0.38, 0.36 | 0.36, 0.26, 0.24 | 0.64, 0.64, 0.69 | 0.65 | 1.79 | 60% | 0.79, 0.79, 0.74 | 1.0 | 0.63, 0.62, 0.54 | XSub_Protect_D2_ControlIndivid =   mean(zq14,zq15, zq16); |
| **Domain3**: **Support Protection** |  |  |  |  |  |  |  |  |  |  |  |  |
| Parental support | 4, 4pt Likert items q21, q22, q23, q24 | 0.60 | 0.31, 0.35, 0.24 0.28, 0.32, 0.15 | 0.18,0.28, 0.15 0.19, 0.26, 0.02 | 0.67, 0.67, 0.66, 0.67 | 0.67 | 1.83 | 46% | 0.72, 0.72, 0.66, 0.60 | 1.0 | 0.51, 0.52, 0.43, 0.36 | XSub_Protect_D3_supportParent =   mean(zq21, zq22, zq23, zq24) ; |
| Parent, teacher, close friend support | q21, q22, q23, q24 (4pt Likert) &  q25, q26 (3pt Likert) | 0.50 | 0.00 to 0.35 | 0.01 to 0.29 | 0.68, 0.69, 0.67 0.76, 0.44 | 0.69 | 1.90 1.04 | 32% 17% | F1:.0.70, 0.71, 0.66, 0.57  F2: 0.29, 0.88 | 1.0 | 0.49, 0.51, 0.48, 0.46 0.22, 0.78 | XComposit_Protect_D3_Support =  mean(XSub_Protect_D3_SupportParent, zq25, zq26); |

[a] Standardize Coefficient Alpha (criterion>0.60), [b] MSA=Kaiser Measure of Sampling Adequacy (Criterion: >5) difference between original correlations & partial correlations, [c] Eigenvalue (criterion > 1.0), [d] Percent of variance explained by a Factor (criterion:> 0.49, min = 0.36), [e] Factor loading: correlations between item & factor (criterion: > 0.70, min 0.60), [f] Final Commonality: % of variance in item explained by Factor, (i.e., factor loading)
